# A quantitative real time PCR method to analyze T cell receptor Vβ subgroup expansion by staphylococcal superantigens

**DOI:** 10.1186/1479-5876-8-2

**Published:** 2010-01-13

**Authors:** Keun Seok Seo, Joo Youn Park, David S Terman, Gregory A Bohach

**Affiliations:** 1 Department of Microbiology, Molecular Biology and Biochemistry, University of Idaho, Moscow, ID 83844, USA; 2 Department of Veterinary Medicine, Washington State University, Pullman, WA 99164, USA; 3 Jenomic, Inc, Carmel, CA, USA

## Abstract

**Background:**

Staphylococcal enterotoxins (SEs), SE-like (SEl) toxins, and toxic shock syndrome toxin-1 (TSST-1), produced by *Staphylococcus aureus*, belong to the subgroup of microbial superantigens (SAgs). SAgs induce clonal proliferation of T cells bearing specific variable regions of the T cell receptor β chain (Vβ). Quantitative real time PCR (qRT-PCR) has become widely accepted for rapid and reproducible mRNA quantification. Although the quantification of Vβ subgroups using qRT-PCR has been reported, qRT-PCR using both primers annealing to selected Vβ nucleotide sequences and SYBR Green I reporter has not been applied to assess Vβ-dependent expansion of T cells by SAgs.

**Methods:**

Human peripheral blood mononuclear cells were stimulated with various SAgs or a monoclonal antibody specific to human CD3. Highly specific expansion of Vβ subgroups was assessed by qRT-PCR using SYBR Green I reporter and primers corresponding to selected Vβ nucleotide sequences.

**Results:**

qRT-PCR specificities were confirmed by sequencing amplified PCR products and melting curve analysis. To assess qRT-PCR efficiencies, standard curves were generated for each primer set. The average slope and R^2 ^of standard curves were -3.3764 ± 0.0245 and 0.99856 ± 0.000478, respectively, demonstrating that the qRT-PCR established in this study is highly efficient. With some exceptions, SAg Vβ specificities observed in this study were similar to those reported in previous studies.

**Conclusions:**

The qRT-PCR method established in this study produced an accurate and reproducible assessment of Vβ-dependent expansion of human T cells by staphylococcal SAgs. This method could be a useful tool in the characterization T cell proliferation by newly discovered SAg and in the investigation of biological effects of SAgs linked to pathogenesis.

## Background

The α/β T cell receptor (TCR) is composed of α and β chain heterodimers which recognize antigen-derived peptide bound to major histocompatibility complex (MHC) molecules on antigen presenting cells (APCs) [1]. During thymocyte development, the genes encoding the β chain undergo somatic recombination of variable (V), diversity (D), joining (J), and constant (C) genes. Combinatorial joining of V-J and V-D-J region gene segments generates diversity within the TCR β chain complementarity determining region (CDR) 3 loop [2,3]. Combinatorial diversity is further increased by imprecise joining of VDJ recombination and insertion of palindromic nucleotides at a specific point within the VD, DJ, and VJ junctions [4]. As a result, each T cell clone expresses a unique variable region of TCR β chain (Vβ) [5]. Generally, the CDR1 and CDR2 sequences within the TCR molecule, encoded by V gene segments, interact with the α helix of the MHC molecule [6]. TCR CDR3 sequences, encoded by V(D)J junction gene segments, interact with the antigenic peptide associated with MHC, resulting in clonal T cell proliferation [6].

Staphylococcal enterotoxins (SEs), SE-like (SEl) toxins and toxic shock syndrome toxin-1 (TSST-1), produced by *Staphylococcus aureus*, are prototypic microbial superantigens (SAgs). Members of this toxin subgroup are implicated in staphylococcal food poisoning and toxic shock syndrome [7]. SEl toxins have been shown to lack emetic properties in primates or have not yet been tested [8]. For many years, five antigenically distinct classic SEs (SEA, SEB, SEC, SED, and SEE) and molecular variants of SEC (SEC1, SEC2, and SEC3) were recognized [7]. Through improvements in genomic analysis tools, novel SEs and SEl toxins including SEG, SElH, SEI, SElJ, SElK, SElL, SElM, SElO, SElP, SElQ, SElR, and SElU and four molecular variants (SEGv, SEIv, SElNv, and SElUv) have been discovered [7,9]. In contrast to conventional antigens, most SAgs bind outside the peptide binding groove of MHC II, and to specific Vβ sequences [9]. This interaction triggers an activation of phospholipase C and phosphokinase C pathways [10], leading to a massive production of proinflammatory cytokines including interleukin-2 and interferon-γ [11], resulting in extensive proliferation of T cells bearing specific Vβ subgroups [11]. As a result, it is possible to characterize SAgs on the basis of their Vβ profiles [7].

Several approaches are used to quantify the expansion of Vβ subgroups including northern blotting, semi-quantitative PCR using radioisotope conjugated probes [12], or fluorescence activated cell sorting (FACS) using monoclonal antibodies (mAbs) specific to Vβ subgroups [13,14]. Recently, quantitative real time PCR (qRT-PCR) has become widely accepted for rapid and reproducible quantification of gene expression. Most previous attempts to quantify Vβ expression using qRT-PCR used one primer located at the gene encoding TCR constant region of β chain (Cβ) and the other primer or fluorogenic probe located within the gene encoding the V region [15,16]. More importantly, previous qRT-PCR methods have been applied to samples displaying expansion of limited numbers of Vβ subgroups [16]. In this study, we developed a new qRT-PCR method using Vβ subgroup specific primers within the gene encoding the V region to increase specificity and SYBR Green I to curtail the cost of the assay. This technique was applied to human mononuclear cell cultures stimulated with various SAgs, which have unique Vβ specificities, though overlapping so that the entire repertoire of Vβ subgroups could be evaluated using this method.

## Materials and methods

### Toxin production and purification

SEB, SEC1 and TSST-1 were purified from cultures of *S. aureus *MNHOCH, *S. aureus *RN4220 (pMIN121) and *S. aureus *RN4220 (pCE107), respectively, using preparative isoelectric focusing as described previously [17-19]. Other toxins used in this study were produced in recombinant form using SE genes cloned in this study as follows. A DNA fragment encoding SEA, SED, SEE, SEG, SEI, SElM, SElN, or SElO was amplified from genomic DNA derived from *S. aureus *FRI 913 or FRI 472 using primers listed in Table 1[20]. Amplified DNA fragments were digested with *NdeI *and *BamHI *or *XhoI *and *ligated *into corresponding sites is pET-15b (Novagen, San Diego, California, USA). Recombinant SE proteins were expressed in *E. coli *BL21 (DE3) (pLysS) and purified using the His-Bind Purification Kit (Novagen) as suggested by the manufacturer.

**Table 1:** List of primers used to clone SE and SEl genes.

SE name	GenBank access number	Forward primer ('5 to 3')	Reverse primer ('5 to 3')
SEA	M18970	cttgtacatatgagcgagaaaagcgaagaa	gcgcggatccttaacttgtatataaata
SED	M28521	cgttctcgagaatgaaaacattgattc	cgcgctcgagctacttttcatataaata
SEE	M21319	ggtagccatatgagcgaagaaataaatgaa	gcgcggatcctcaagttgtgtataaata
SEG	AF064773	tgtgcatatgcaacccgatcctaaatta	gcgcggatcctcagtgagtattaaga
SEI	AF285760	tgctctcgaggatattggtgtaggtaac	cgcgctcgagttagttactatctacata
SElM	AF285760	cgcacatatggatgtcggagttttgaat	gcgcggatcctcaactttcgtccttata
SElN	AF285760	aatgctcatatggacaaaaaagatttaaag	gcgcggatccttaatctttatataaaa
SElO	AF285760	tgcactcgagaatgaagaagatcctaaa	cgcgctcgagttatgtaaataaataaac

### Preparation and stimulation of enriched human lymphocytes

Peripheral blood mononuclear cells (PBMCs) were isolated from three healthy donor venous blood. Heparin-treated (14 U/ml blood) blood was fractionated by gradient centrifugation over Ficoll-Paque Plus (GE Healthcare, Piscataway, New jersey, USA) as described previously [17]. The PBMCs were washed and resuspended in RPMI 1640 medium (Life technologies, Gaithersburg, Maryland, USA) supplemented with 2% FBS, 100 U penicillin G, and 100 μg/ml streptomycin. The cultures were maintained in cell culture Petri dishes (Falcon, Lincoln Park, New Jersey) overnight at 37°C and in 5% CO_2_. Non-adherent lymphocyte-enriched PBMCs were collected, washed, and resuspended at a final concentration of 2.5 × 10^6 ^cells/ml. Each SAg (0.5 μg/ml) or a murine mAb specific to human CD3 (33 ng/ml; Sigma, St. Louis, Missouri, USA) was added to lymphocyte enriched PBMC cultures (3 ml aliquots). Cultures were maintained for 4 days (37°C, 5% CO_2_). Basal levels of Vβ expansion were assessed with unstimulated control cultures.

### Quantitative RT-PCR (qRT-PCR)

Total RNA was extracted from approximately 5 × 10^6 ^cells using Trizol (Life Technologies). Superscript II reverse transcriptase (Life Technologies) was used to generate cDNA using 1 μg of RNA and oligo dT primer, according to the manufacturer's instructions. To promote highly specific amplification, two primers specific for each of the various Vβ subgroups were annealed to selected Vβ nucleotide sequences. All Vβ specific and Cβ primers were designed using Primer Express version 2.0 (Applied Biosystems, Foster City, California, USA) and are listed in Table 2. We used the Vβ subgroup nomenclature of Arden et al [21].

**Table 2:** List of qRT-PCR primersa and amplified Vβ gene(s).

Primer name	GenBank access number	Forward primer ('5 to 3')	Reverse primer ('5 to 3')	Amplified Vβ gene(s)^b^
Cβ	L36092	tccagttctacgggctctcg	gacgatctgggtgacgggt	
VB1	L36092	ggagcaggcccagtggat	cgctgtccagttgctggtat	TCRVB1s1
VB2	M11955	gagtctcatgctgatggcaact	tctcgacgccttgctcgtat	TCRVB2s1
VB3	U08314	tcctctgtcgtgtggccttt	tctcgagctctgggttactttca	TCRVB3s1
VB4	L36092	ggctctgaggccacatatgag	ttaggtttgggcggctgat	TCRVB4s1
VB5	L36092	gctccaggctgctctgttg	tttgagtgactccagcctttactg	TCRVB5s1, 5s3
VB6	X61440	ggcagggcccagagtttc	gggcagccctgagtcatct	TCRVB6s1, 6s2, 6s3, 6s4, 6s5, 6s6
VB7	U07977	aagtgtgccaagtcgcttctc	tgcagggcgtgtaggtgaa	TCRVB7s1, 7s2, 7s3
VB8	X07192	tgcccgaggatcgattctc	tctgagggctggatcttcaga	TCRVB8s1, 8s2, 8s3
VB9	U07977	tgcccgaggatcgattctc	tctgagggctggatcttcaga	TCRVB9s1
VB11	L36092	catctaccagaccccaagatacct	atggcccatggtttgagaac	TCRVB11s1
VB12	U03115	gttcttctatgtggccctttgtct	tcttgggctctgggtgattc	TCRVB12s1, 12s3
VB13A	L36092	tggtgctggtatcactgaccaa	ggaaatcctctgtggttgatctg	TCRVB13s1, 13s6
VB13B	X61445	tgtgggcaggtccagtga	tgtcttcaggacccggaatt	TCRVB13s2, 13s9
VB14	L36092	gctccttggctatgtggtcc	ttgggttctgggtcacttgg	TCRVB14s1
VB15	M11951	tgttacccagaccccaagga	tgacccttagtctgagaacattcca	TCRVB15s1
VB16	X06154	cggtatgcccaacaatcgat	caggctgcaccttcagagtaga	TCRVB16s1
VB17	U48260	caaccaggtgctctgctgtgt	gactgagtgattccaccatcca	TCRVB17s1
VB18	L36092	ggaatgccaaaggaacgattt	tgctggatcctcaggatgct	TCRVB18s1
VB20	L36092	aggtgccccagaatctctca	ggagcttcttagaactcaggatgaa	TCRVB20s1
VB21	M33233	gctgtggctttttggtgtga	caggatctgccggtaccagta	TCRVB21s1
VB22	L36092	tgaaagcaggactcacagaacct	tcacttcctgtcccatctgtgt	TCRVB22s1
VB23	U03115	ttcagtggctgctggagtca	cagagtggctgtttccctcttt	TCRVB23s1
VB24	U03115	acccctgataacttccaatcca	cctggtgagcggatgtcaa	TCRVB24s1

^a^The pseudogenes (Vβ10 and Vβ19) were not included in this study.

^b^Vβ subgroup nomenclature followed the classification of Arden et al. [21].

To verify primer specificities, melting curve analyses (below) and PCR product sequencing were performed. For sequencing, PCR reactions were conducted without SYBR Green I using cDNA generated from cultures stimulated CD3-specific mAb. PCR products were purified using a PCR purification kit (Qiagen, Valencia, California, USA) and then cloned into pCR2.1 vector (Life Technologies). Transformants (10 to 25 colonies) were randomly selected and the cloned gene fragments were sequenced using an ABI Prism 3100 Genetic Analyzer (Applied Biosystems).

Standard curves were generated for each gene to evaluate primer efficiency and for data analysis. Concentrations of purified PCR products were determined by measuring the absorbance at 260 nm using a Nanodrop (Thermo Scientific, Wilmington, Delaware, USA) and expressed as the number of DNA copies/ml using standard procedures [22,23]. The qRT-PCR was performed (below) on serially diluted PCR products (2.5 - 2.5 × 10^5 ^copies/reaction) using ABI Prism 7500 (Applied Biosystems) in triplicate and was repeated in at least three separate experiments. Standard curves were generated by plotting the C_T _vs. the log_10_^copies ^of serially diluted PCR products. The slope, intercept, and correlation coefficient (R^2^) were determined by linear regression analysis using Microcal OriginPro Version 7.5 (OriginLab, Northampton, Massachusetts, USA).

The qRT-PCR was performed in triplicate and was repeated in at least three separate experiments using the following conditions. Reaction mixtures contained 12.5 μl of SYBR Green I dye master mix (Applied Biosystems), 2 pmoles each of forward and reverse primers, and 5 μl of 100 times diluted cDNA. Thermocycle conditions included initial denaturation at 50°C and 95°C (10 min each), followed by 40 cycles at 95°C (15 s) and 60°C (1 min). Fluorescent data were acquired during each extension phase. After 40 cycles, a melting curve was generated by slowly increasing (0.1°C/s) the temperature from 60°C to 95°C, while the fluorescence was measured. The threshold cycle (C_T_) was calculated using the Sequence Detector Systems version 1.2.2 (Applied Biosystems) by determining the cycle number at which the change in the fluorescence of the reporter dye (ΔRn) crossed the threshold. To synchronize each experiment, the baseline was set automatically by the software. To rule out DNA contamination in the RNA preparations, the qRT-PCR controls were performed with RNA templates which did not show any amplification.

### Data analysis

Calculations to determine the extent of Vβ expansion were done as described by calculating the absolute copy number of each Vβ and Cβ. Briefly, the C_T _for each Vβ and Cβ was converted into absolute copy number by extrapolation from its standard curve (above). The percentage of each Vβ (%Vβ) in the culture was calculated by following equation, where n represents each Vβ subgroup observed in this study. These values have to be considered as exploratory:

Selective expansion of Vβs in the culture stimulated with SAgs was determined when each %Vβ from the cultures stimulated with SAgs was significantly higher than the corresponding %Vβ from the control cultures (without stimuli) by paired *t*-test (p < 0.001) using SAS statistical software (version 9.0, SAS Institute Inc., Cary, North Carolina, USA).

## Results

### Sensitivity and efficiency of the qRT-PCR

**Figure 1:**
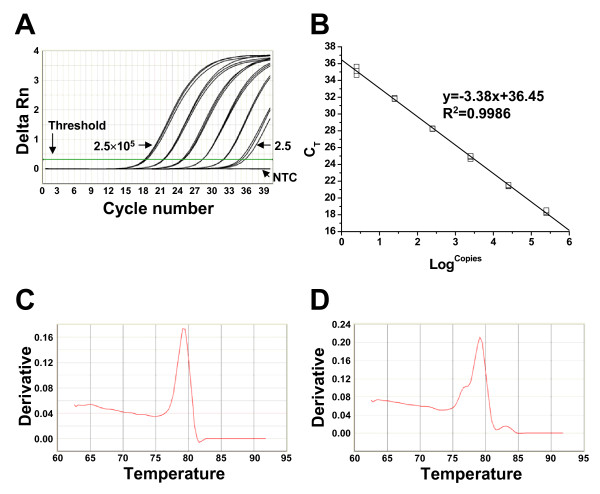
**The specificity, sensitivity, and reproducibility of qRT-PCR**. The qRT-PCR was performed using a ten-fold dilution series (2.5 × 10^5 ^to 2.5 copies/reaction) of purified PCR product. Results shown are from a single representative experiment that was conducted three times. (A) The qRT-PCR successfully amplified the ten-fold dilution series of template (2.5 × 10^5 ^to 2.5 copies/reaction; from left to right). The non-template control (NTC) showed no amplification. The threshold was automatically set by Sequence Detector Systems version 1.2.2 software to synchronize among experiments. The threshold cycle (C_T_) was determined by the cycle number at which the change in the fluorescence of the reporter dye (delta Rn) crossed the threshold; (B) The standard curve was generated by plotting the C_T _vs the number of purified PCR product copies (Log^copies^). The slope and correlation coefficient (R^2^) were -3.38 and 0.9986, respectively; (C) Melting curve analysis for the Vβ1 subgroup consist of single subgroup gene and showed a single peak at 78 oC; (D) Melting curve analysis for the Vβ17 subgroup consisting of three subgroup genes showed multiple peaks, consistent with the expected heterogeneity among amplified products.

cDNA was generated from cultures stimulated with a CD3-specific mAb, amplified by PCR using primers specific for Vβ, Cβ, and G3PDH genes. Standard curves were generated using the purified PCR products. Representative results from a Cβ primer-based reaction are shown in Figure 1A. The qRT-PCR could detect ≤25 copies of Cβ PCR products without detectable variation among triplicate reactions (Figure 1A). The slope and correlation coefficient (R^2^) of standard curves are used to determine primer efficiency and standard curve validity, respectively. Results obtained with the Cβ reaction are representative data showing the slope for Cβ reaction was -3.38 with R^2 ^value of 0.9986 (Figure 1B). The slope and R^2 ^value of standard curves for other reactions are listed in Table 3.

**Table 3:** Standard curve slopes, Y axis intercepts and correlation coefficients (R^2^)

Primers	Slope	Y axis intercept	Correlation coefficient (R^2^)
Cβ	-3.38	36.45	0.9986
VB1	-3.39	36.54	0.9977
VB2	-3.36	36.38	0.9982
VB3	-3.41	36.57	0.9987
VB4	-3.37	36.62	0.9984
VB5	-3.35	36.33	0.9976
VB6	-3.40	36.53	0.9978
VB7	-3.36	36.43	0.9983
VB8	-3.37	36.40	0.9986
VB9	-3.38	36.49	0.9985
VB11	-3.41	36.52	0.9986
VB12	-3.42	36.53	0.9972
VB13A	-3.34	36.34	0.9978
VB13B	-3.41	36.54	0.9974
VB14	-3.36	36.33	0.9981
VB15	-3.35	36.44	0.9976
VB16	-3.37	36.44	0.9984
VB17	-3.39	36.53	0.9982
VB18	-3.35	36.44	0.9986
VB20	-3.33	36.39	0.9973
VB21	-3.36	36.38	0.9986
VB22	-3.39	36.47	0.9981
VB23	-3.37	36.43	0.9980
VB24	-3.41	36.53	0.9984

### Specificity of the qRT-PCR

qRT-PCR specificities were assessed by melting curve analysis and sequencing of amplified PCR products. Melting curves of the qRT-PCR reactions observed for Vβ subgroups consisting of a single gene, as well as G3PDH and Cβ genes, showed single peak. A representative result obtained for Vβ1 is shown in Figure 1C. The melting curve for the Vβ1 reaction contained a single peak at 78°C. Melting curves for Vβ subgroups consisting of multiple subgroup genes (Vβ7, 12, 13A, and 17) showed multiple peaks due to the expected heterogeneity in amplified gene fragments. Representative results for Vβ17 are shown in Figure 1D. qRT-PCR for Vβ17, which contains three subgroup genes produced a melting curve with three peaks as expected. However, some Vβ subgroups consisting of multiple subgroup genes (Vβ5, 6, 8, 13B, and 21) showed only a single peak (data not shown). To identify whether primers designed for these Vβ subgroups amplified the targeted subgroup genes, PCR products were cloned and sequenced. This revealed that, except for Vβ21, the primers amplified multiple Vβ subgroup genes within the targeted Vβ subgroup (Table 2). No untargeted sequences were generated. We also analyzed the amplified PCR products using agarose gel electrophoresis and confirmed that there was no non-specific amplification other than expected size of amplification product (data not shown).

### Quantification of Vβ expansion

**Figure 2:**
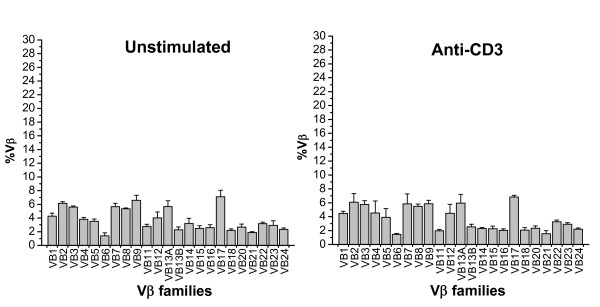
**Vβ subgroup representation (%Vβ) in unstimulated cultures and in cultures stimulated with a CD3-specific mAb**. %Vβs (mean ± S.E.M.) in cultures prior to stimulation or cultures of the same cell preparations after four days in the presence of the mAb. There was no significant differences in %Vβs calculated for either condition (p < 0.05). Results shown are the mean ± S.E.M. of three sets of triplicates combined from three experiments (n = 9).

**Figure 3:**
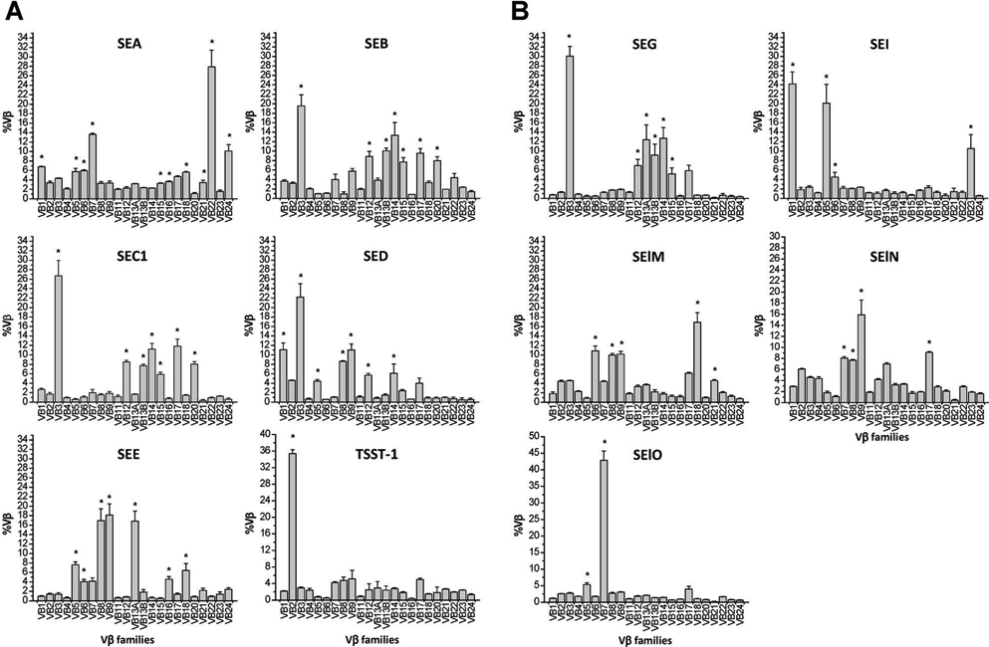
**Distribution of %Vβ in the cultures stimulated with SAgs**. Non-adherent lymphocyte-enriched PBMCs were stimulated with SAgs (final concentration at 5 μg/ml) for 4 days. The %Vβs were calculated and were presented as the mean ± S.E.M. Asterisks indicate a significant increase in %Vβ compared to cultures without stimuli (p < 0.05). Results shown are the means ± S.E.M. of three sets of triplicates combined from three experiments (n = 9). A) Classic SEs and TSST-1. B) Novel SEs and SEls.

To assess the basal expression level, the percentage of each Vβ (%Vβ) in cultures without stimuli was calculated (Figure 2; unstimulated panel). Similarly, the expression of each Vβ subgroup gene was determined for cultures stimulated with anti-CD3 mAb or various SAgs. Selective expansion of T cells bearing certain Vβ subgroups was considered to be significant when the %Vβ in the stimulated cultures was elevated at a statistically significant level (p < 0.05). There was no significant difference among levels observed in cultures stimulated with the anti-CD3 mAb and unstimulated cultures (Figure 2). In contrast, the pattern of Vβ expression in cultures stimulated with various SAgs showed a distinct expansion of T cells bearing certain Vβ subgroups (Figures 3, Table 4). The data indicate that each Vβ subgroup was expanded by one or more SAgs used in this study. As shown in Table 4, the Vβ specificities of SAgs observed in this study were very similar to those described in previous studies with minor variation as discussed below [7,11,12,24-26].

**Table 4:** Comparison of Vβ specificity observed in this study with those in selected previous studies.

SAgs	Vβ specificity observed in this study	Vβ specificity observed in previous studiesa	References
SEA	Vβ1, 5, 6, 7, 15, 16, 18, 21, 22, 24	Vβ1, 5, 6, 7, 9, 16, 18, 21	[24]
SEB	Vβ3, 12, 13B^b^, 14, 15, 17, 20	Vβ1, 3, 6, 12, 13.2, 15, 17, 20	[11]
SEC1	Vβ3, 12, 13B, 14, 15, 17, 20	Vβ3, 12, 13.2, 14, 15, 17, 20	[12]
SED	Vβ1, 3, 5, 8, 9, 12, 14	Vβ1, 5, 6, 7, 8, 12	[7,25]
SEE	Vβ5, 6, 8, 9, 13Ac, 16, 18	Vβ5, 6, 8, 13.1, 18, 21	[11,24]
SEG	Vβ3, 12, 13A, 13B, 14, 15	Vβ3, 12, 13, 14	[26]
SEI	Vβ1, 5, 6, 23	Vβ1, 5, 6, 23	[26]
SElM	Vβ6, 8, 9, 18, 21	Vβ6, 8, 9, 18, 21	[26]
SElN	Vβ7, 8, 9, 17	Vβ9	[26]
SElO	Vβ5, 7	Vβ5, 7, 22	[26]
TSST-1	Vβ2	Vβ2	[11]

^a^Vβ specificities were results from previous studies using semi-quantitative PCR or FACS methods.

^b^Vβ13B corresponds to Vβ13.2 in previous studies.

^c^Vβ13A corresponds to Vβ13.1 in previous studies.

## Discussion

More than 67 different human Vβ genes, of which a quarter are pseudogenes, have been have been cloned and sequenced [2,21,27]. These studies confirmed the existence of 49 functional Vβ genes within 24 different Vβ subgroups. Due to the heterogeneity, some of the 24 Vβ subgroups consist of multiple subgroup genes. In this study, we designed two primers annealing to each of 22 different Vβ subgroups (36 Vβ genes) to quantify expansion of T cell bearing specific Vβ subgroups and subgroup genes in response to SAgs.

One of the important factors that affect the validity of qRT-PCR is the efficiency of primers. The primers used in qRT-PCR should have uniform and high efficiency to achieve a valid quantification. The efficiency and linearity of primers could be assessed by analyzing the slope and R^2 ^value of the standard curve, respectively. In theory, the slope should be close to -3.32 with an optimal efficiency when 10-fold serially diluted templates were used. The average slope and R^2 ^of standard curves for all primers used in qRT-PCR was -3.3764 ± 0.0245 and 0.99856 ± 0.000478, respectively. This suggests that all primers used in qRT-PCR have uniform and high efficiency and linearity.

The specificity of qRT-PCR using SYBR Green I platform was often determined by analyzing melting curves. In this study, the specificities of each primer set were determined by analyzing melting curves and sequencing amplified PCR products. Melting curve analysis and sequencing amplified PCR products of reactions for, Cβ and Vβ subgroups consisting of a single subgroup showed a single peak and a single specific amplification. As expected, some Vβ subgroups comprised of multiple subgroup genes (Vβ7, 12, 13A, and 17) showed a corresponding number of peaks. However, some Vβ subgroups comprised of multiple subgroup genes (Vβ5, 6, 8, 13B, and 21) showed only a single peak. The sequence analysis of amplified PCR products for Vβ5, 6, 8, 13B, and 21 subgroups revealed that multiple subgroup genes were amplified. For example, the Vβ6 subgroup, consisting of 6 functional subgroup genes with > 87.9% sequence similarity to each other, showed a single peak in melting curve analysis, though the sequence analysis of amplified PCR product showed that all 6 functional subgroup genes were amplified. The resolution of these into a single peak probably due to a high level nucleotide sequence similarity among subgroup genes resulting in an identical meting temperature of amplified gene fragments. The identity of all sequenced PCR products matched with corresponding subgroups of Vβ subgroups and revealed that 36 out of 49 functional Vβ subgroup genes were amplified. It suggests that primers used in this study were highly specific to targeted Vβ subgroup.

In this study, we used various SAgs showing similar and/or unique Vβ specificities covering the entire repertoire of human Vβ subgroups. The qRT-PCR showed that every Vβ subgroup was expanded in this study. As shown in Table 4, the Vβ specificities of SAgs observed in this study was very similar to those described in previous studies with minor variation [7,11,12,24-26]. In this study, newly identified Vβ specificities were observed for some SAgs such as SEA (Vβ15, 22, and 24), SEB (Vβ 14), SED (Vβ3, 9, and 14), SEE (Vβ9 and 16), SEG (Vβ15), and SElN (Vβ7, 8, and 17). Also, some Vβ previously reported specificities were not observed for some SAgs such as SEB (Vβ1 and 6), SED (Vβ6 and 7), SEE (Vβ21), and SElO (Vβ22). These discrepancies might be explained by the differences in the repose to SAgs among humans or differences in techniques (PCR, flow cytometry), or the lack of reagents at the time of previous studies. For example, the Vβ specificity of some SAgs in two previous studies was determined by semi-quantitative PCR using primers specific to Vβ1 through Vβ20 [11,12]. This present study incorporated primers specific to Vβ21 through Vβ24. However, it is noteworthy that Vβ subgroups most prominently expanded by each SAg observed in this study were identical to those observed in previous studies.

## Conclusion

In this report, we developed an assay to quantify the expansion of human Vβ subgroups using qRT-PCR. The specificity and efficiency of the method were evaluated by generating standard curves for each primer set. The validity of the method was assessed by analyzing the Vβ specificity of various SAgs which combined, interact with Vβ repertoires covering all known Vβ subgroups. Our results demonstrate that the method established in this study is accurate, sensitive, and highly reproducible. This qRT-PCR method could also be used to characterize novel SAgs, to determine complete profiles of currently known SAgs, and to help understand the role of T cells bearing specific Vβs in certain diseases such as neoplastic expansion of large granular lymphocytes, T cell non-Hodgkin's lymphoma [28,29] as well as some immune disorders associated with SAgs such as immunosuppression, Kawasaki disease, and atopy [30-32].

## Competing interests

The authors declare that they have no competing interests.

## Authors' contributions

KSS developed the basic assay and performed most experiments including cloning, protein purification, cell preparation and stimulation, qRT-PCR, and data analysis. JYP helped to perform qRT-PCR and interpret data. DST provided some toxins and input into general experimental strategy. GAB assisted in experimental design and helped to interpret data and draft the manuscript. All authors read and approved the final manuscript.
